# Soil Texture and Cultivar Effects on Rice (*Oryza sativa*, L.) Grain Yield, Yield Components and Water Productivity in Three Water Regimes

**DOI:** 10.1371/journal.pone.0150549

**Published:** 2016-03-15

**Authors:** Fugen Dou, Junel Soriano, Rodante E. Tabien, Kun Chen

**Affiliations:** 1 Texas A&M AgriLife Research Center at Beaumont, 1509 Aggie Dr., Beaumont, TX, 77713, United States of America; 2 International Crops Research Institute for the Semi-Arid Tropics, Patancheru 502 324, Telangana, India; 3 Department of Statistics, University of Connecticut, Storrs, CT, 06269, United States of America; Tennessee State University, UNITED STATES

## Abstract

The objective of this study was to determine the effects of water regime/soil condition (continuous flooding, saturated, and aerobic), cultivar (‘Cocodrie’ and ‘Rondo’), and soil texture (clay and sandy loam) on rice grain yield, yield components and water productivity using a greenhouse trial. Rice grain yield was significantly affected by soil texture and the interaction between water regime and cultivar. Significantly higher yield was obtained in continuous flooding than in aerobic and saturated soil conditions but the latter treatments were comparable to each other. For Rondo, its grain yield has decreased with soil water regimes in the order of continuous flooding, saturated and aerobic treatments. The rice grain yield in clay soil was 46% higher than in sandy loam soil averaged across cultivar and water regime. Compared to aerobic condition, saturated and continuous flooding treatments had greater panicle numbers. In addition, panicle number in clay soil was 25% higher than in sandy loam soil. The spikelet number of Cocodrie was 29% greater than that of Rondo, indicating that rice cultivar had greater effect on spikelet number than soil type and water management. Water productivity was significantly affected by the interaction of water regime and cultivar. Compared to sandy loam soil, clay soil was 25% higher in water productivity. Our results indicated that cultivar selection and soil texture are important factors in deciding what water management option to practice.

## Introduction

Water as a natural resource is becoming limiting in production agriculture. Drought has been reported in several countries affecting their food production [1–2]. With climate change, this problem can be aggravated thus water has to be used efficiently. Efficiency in water management is commonly measured by water productivity (WP), defined as the ratio of the marketable crop yield over actual evapotranspiration [3]. In the past decades, rice WP has increased substantially. A recent review has indicated that rice WP has more than doubled in the past 20 years from an average of around 0.34 g paddy rice per kg water to around 0.77 g kg^-1^ [3], largely due to increased yield from the development and adoption of improved varieties and management strategies [4], and to a lesser degree to the introduction of rice water management [5].

In addition, soil can play important roles in rice production in terms of water productivity. First, soil texture can affect soil available water capacity (AWC). Usually, clay soil contains more organic matter than sandy soil because of greater physical protection attributed from clay [6]. Greater content of organic matter generally means greater AWC. After a critical review, Hudson [7] reported that as soil organic matter content increased from 0.5 to 3%, AWC of the soil is more than doubled. Loss of organic matter coupled with soil compaction can significantly reduce crop yield [8]. Secondly, soil also affects crop root growth, a main organ in water uptake. In particular, soil texture or structure can affect root production. Usually, bigger roots have greater potential in elongation and therefore can enhance better water and nutrient uptake, and overall root production. Root growth of the same cultivar can vary with soil texture. Therefore, it is critical to determine the impact of soil properties on different production systems related to water regime along with rice cultivar. The objective of this study was to assess the effects of water regimes, rice cultivar, and soil texture on rice grain yields, yield components and water productivity in a greenhouse trial.

## Material and Methods

The pot experiment was established at the greenhouse of the Texas A&M Agrilife Research Center at Beaumont, Texas and conducted from August 2011 to February 2012. A factorial experimental design composed of three factors namely; water management, rice cultivar and soil texture was used with three (3) replications. Water management had three water regimes; *aerobic soil condition*, *saturated soil condition* and *flooded soil condition*. Two soils and rice cultivars were used with a complete randomization, *clay soils (S*_*1*_*)* from Beaumont Center and *sandy loam soils (S*_*2*_*)* taken at Eagle Lake station. The soil at Beaumont was a League clay soil (fine, montmorillonitic, Entic Pelludert) and the soil at Eagle Lake was a Hockley silt loam (fine, smectitic, hyperthermic Typic Albaqualfs). The main soil properties were listed in Table 1. Briefly, soil texture was measured using a hydrometer procedure [9]. A 1:2 soil: water extract was used to measure soil pH [10]. Soil samples were oven dried at 105°C and finely ground to measure SOC by combustion using an Elementar Americas Inc, Vario MAX CN analyzer (Mt. Laurel, NJ, U.S.A) [11]. Soil nitrate was extracted by a 1 M KCl solution and determined by a Cd-reduction method [12]. Other plant available elements including P, K, Ca, Mg, Na and S were extracted using the Mehlich III extractant and were determined by ICP [13]. Two rice cultivars were *Rondo (V*_*1*_*)* and *Cocodrie (V*_*2*_*)*. Cocodrie was bred by the Louisiana Rice Research Station at Crowley, LA, and very popular in southern US rice belt [14]. As a long-gain *indica* cultivar, Rondo was bred by USDA ARS and had features of high yield potential, an excellent disease resistance package, and premium processing quality [15].

**Table 1:** Main soil properties of the soil samples from Beaumont Center and Eagle Lake Station, Texas.

	Silt (g kg^-1^)	Clay (g kg^-1^)	pH	SOM (g kg^-1^)	Nitrate (mg kg^-1^)	P (mg kg^-1^)	K (mg kg^-1^)	Ca (mg kg^-1^)
Clay	22	64	5.9	12	2	13	267	4716
Sandy Loam	16	15	5.9	7	1	36	48	753

Rondo and Cocodrie were grown in 18-cm high plastic pots with 12-cm and 15-cm bottom and top diameters, respectively. Twelve (12) pots were placed in one wooden box (87 x 87 x 40 cm) lined with black plastic sheet to keep water and avoid water spill during irrigation and throughout the growth period. Water was applied to the plastic-lined wooden box with the pots before seeding. Pre-germinated seeds were sowed manually on top of the wet soils at 3–5 seeds per pot. Thinning was done up to week 3 to maintain one seedling per pot.

Water regimes were imposed starting week 3. In aerobic soil condition, irrigation water was applied to moist the soil near field capacity or when soil suction reading reached at 40 kPa from week 3 to booting stage and from grain filling stage to terminal irrigation or one week before harvest. Floodwater depth of 3–5 cm was maintained at booting to flowering stage in aerobic soil condition. For saturated soil condition, irrigation water level in the wooden box was maintained at 2–5 cm below the soil surface in the pots to continuously saturate the soil until terminal irrigation. Continuous flooding with floodwater depth of 3–5 cm was kept in flooded soil condition from week 3 to terminal irrigation. Terminal irrigation or water from each box was removed 3 days before crop cutting at physiological maturity (PM). The two varieties have different maturity but were put together in each box set for a water regime. However, to facilitate final data gathering, the pots with similar cultivar under the same water regimes were grouped and re-arranged 10 days before terminal irrigation. Irrigation was done using the delivery hose connected into the water system of the greenhouse and the volume of water applied was expressed in cubic meters and calculated based on water discharge, desired water depth, time of irrigation, and area or volume of the box and pots.

In all pots, phosphorous (P) and potassium (K) fertilizers at 67 kg ha^-1^ was applied as basal by incorporating with the soil during soil medium preparation both for the two soils. Nitrogen (N) was applied in week 2, 5 and 8 at the rate of 20%, 50% and 30% with the recommended N, 280 kg ha^-1^ for *S*_*1*_ and 235 kg ha^-1^ for *S*_*2*_, respectively. Hand weeding was done to keep the pots weed-free. Granular insecticides were applied at maximum tillering and flowering stages to avoid panicle mites and other insects infestation.

Grain yield (GY) was determined from a single representative plant. Grains were threshed manually, air dried for two to three days and weighed. Grain moisture content was determined using digital moisture meter after weighing. Grain yield was adjusted to 12% moisture content. Adjusted grain yield was expressed in gram plant^-1^.

Yield components (YC) including panicle number per plant, total number of spikelets per panicle, number of filled spikelets and 500-grain weight at harvest were determined. Grains were threshed from all panicles after oven drying at 40°C for 12 hrs, and weighed. Filled and unfilled grains were separated, counted, weighed and the percent filled spikelet was calculated. Grain weight (g) was obtained in 500 seeds per plant. Water productivity is defined as the amount of filled spikelets or grain produced per unit quantity of water. The water productivity is obtained by dividing the total grain produced in each pot by the total amount of water used.

Analysis of variance (ANOVA) for a factorial experimental design was performed to determine main effects and interaction effects of water regime, cultivar, and soil texture (SAS, 2012). All significant treatment effects were determined using the LSD at *P* < 0.05, and correlation coefficients were calculated at *P* < 0.05.

## Results and Discussion

### Rice Grain Yield

Soils with different texture significantly affected rice grain yields (Tables 2 and 3). The rice grain yield in clay soil was 46% higher than in sandy soil. Clay soil has more fine particles that can hold water and nutrients better than sandy soil, thus it can retain more water and nutrients needed by the water loving rice plant. Conversely, sands provides easier passage through its aggregation, retaining less water including nutrients, thus may not meet the demands of the plants, particularly during the grain development. The same observation was noted in China when varieties were evaluated in clay and sandy soil [16–17] but clay had less yield increase relative to sandy soil in varying nitrogen levels [17]. In a rainfed lowland of Thailand, Tsubo et al. [18] also reported the same response, rice grown in higher clay-content soils had greater grain yield and biomass accumulation than those grown in lower clay content soils.

**Table 2:** Significance of the main effects [water regime (W), cultivar (CV), and soil texture (S)] and interactions among the main effects for rice grain yield and yield components across environments for clay and silt loam soil.

	Rough rice yield	Grain weight	Panicle number	Spikelet number	Filled spikelet	Water productivity
Effect	----------*P* value--------					
Water	0.01	0.20	<0.01	0.15	0.32	<0.01
Soil	<0.01	0.55	<0.01	<0.01	0.64	<0.01
WaterXSoil	0.99	0.34	0.89	0.22	0.06	0.77
Cultivar	0.01	0.14	<0.01	<0.01	0.03	0.50
Water* Cultivar	0.01	0.14	0.02	0.34	0.13	<0.01
Soil* Cultivar	0.99	0.87	0.23	0.78	0.12	0.99
Water*Soil* Cultivar	0.42	0.30	0.49	0.37	0.20	0.15

**Table 3:** The effect of soil texture on rice production.

	Rough rice yield	Grain weight	Panicle number	Spikelet number	Water productivity
Clay	30.8^a^*	10.8	14.6^a^	176.9^a^	0.5^a^
Sandy Loam	21.1^b^	10.9	11.7^b^	149.2^b^	0.4^b^
LSD (α = 0.05)	3.5	0.7	1.5	18	0.05

*Means with the same letter are statistically not different at the same column.

**Fig 1:**
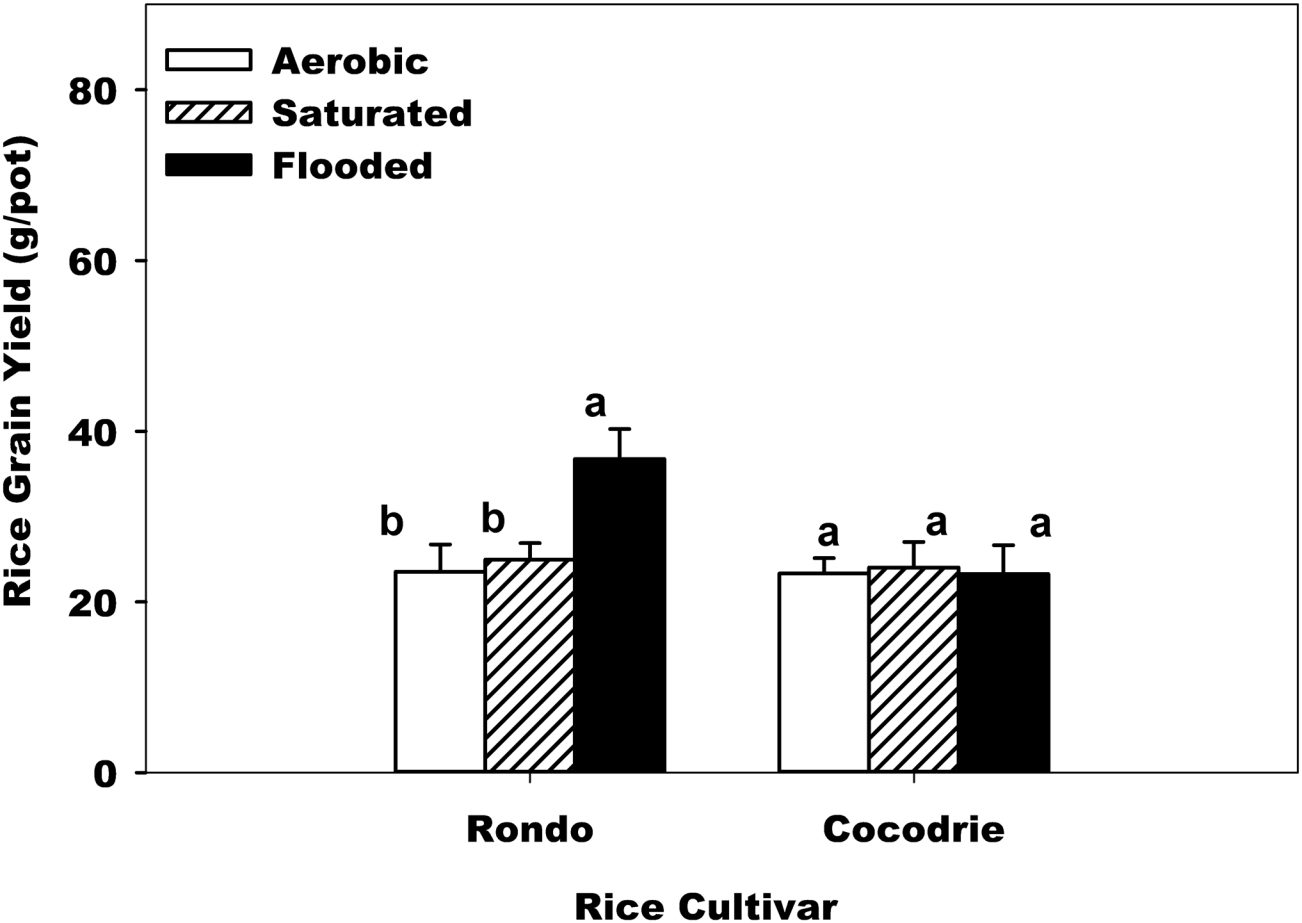
Effect of water regime and cultivar across soil texture on rough rice grain yield (g/pot). Error bars denote the standard error of the mean. Bars with the same letters above are not significantly different based on Fisher’s protected LSD (*P* = 0.05) within the cultivar.

Variation in grain yield of two varieties was found highly significant and this has been the case in series of yield trials done in flooded fields [19]. Rondo was always yielding much higher than Cocodrie. Yield response of the cultivar was found to vary depending on the water regimes. The ANOVA showed highly significant interaction between water regimes and variety (Table 2). For Rondo, grain yield decreased with soil water regimes in the order of continuous flooding, saturated and aerobic treatments (Fig 1). The grain yields of Rondo under aerobic and saturated water regimes were 56% and 47% lower than yield in continuous flooding, respectively. For Cocodrie, however, the highest grain yield was with saturated water regime and lowest with continuous flooding (Fig 1). The highest yield was 37 g per rice crop with Rondo at saturated regime. Changes in water regimes significantly affected rice grain yield of Rondo but not much with Cocodrie, indicating that Cocodrie can be grown in all three water regimes without significant yield losses. Kato et al. [20] reported a cultivar like Cocodrie that had no yield penalty when grown in aerobic condition. Japanese cultivar ‘Takanari’ achieved yields greater than 10 t ha^-1^ when grown under aerobic condition. Similar effect of rice cultivar on grain yield under aerobic system has also been reported [16–21]. Breeding rice for aerobic production system has been recommended since current varieties are all developed for flooded growing condition. Kato et al [20] showed that aerobic rice can out yield current varieties. Recently, Zhao et al. [22] reported that most of tested rice genotypes bred for tropic aerobic conditions out-yielded check varieties, with 10% higher yield and greater harvest index. The increased yield was attributed to greater drought tolerance and harvest index compared to the conventional lowland or upland cultivars. These results suggest the potential of developing rice cultivars appropriate for aerobic production system.

### Rice Grain Weight

Water regime and soil type did not affect the weight of 500 seeds (alternative 1000-grain). Only cultivar had significant effect on rice grain weight (Table 2). The grain weight of Rondo was 12% greater than that of Cocodrie (data not shown). Visually, seeds of Rondo were bolder and thicker while Cocodrie seeds were relatively much smaller and thinner, having typical long grain rice appearance. Yoshida et al. [23] suggested that rice 1000-grain weight is mainly affected by the hull size that is genetically controlled. Fan et al. [24] reported that 1000-grain weight was mainly controlled by a major quantitative locus (QTL), GS3.

For each cultivar, there was no difference in 500-grain weight between aerobic, saturated, and flooded water systems (Table 2) supporting the idea that this trait is not sensitive to change in soil texture and water regimes that avoid water stress. Peng et al. [25] reported that aerobic rice treatment significantly decreased 1000-rice grain weight in seven of eight rice seasons but this could be due to typical aerobic system with rice grown in unsaturated soil. Other management practices including seeding density also do not show effect on 1000-grain weight in Texas [26].

### Rice Panicle Number

**Fig 2:**
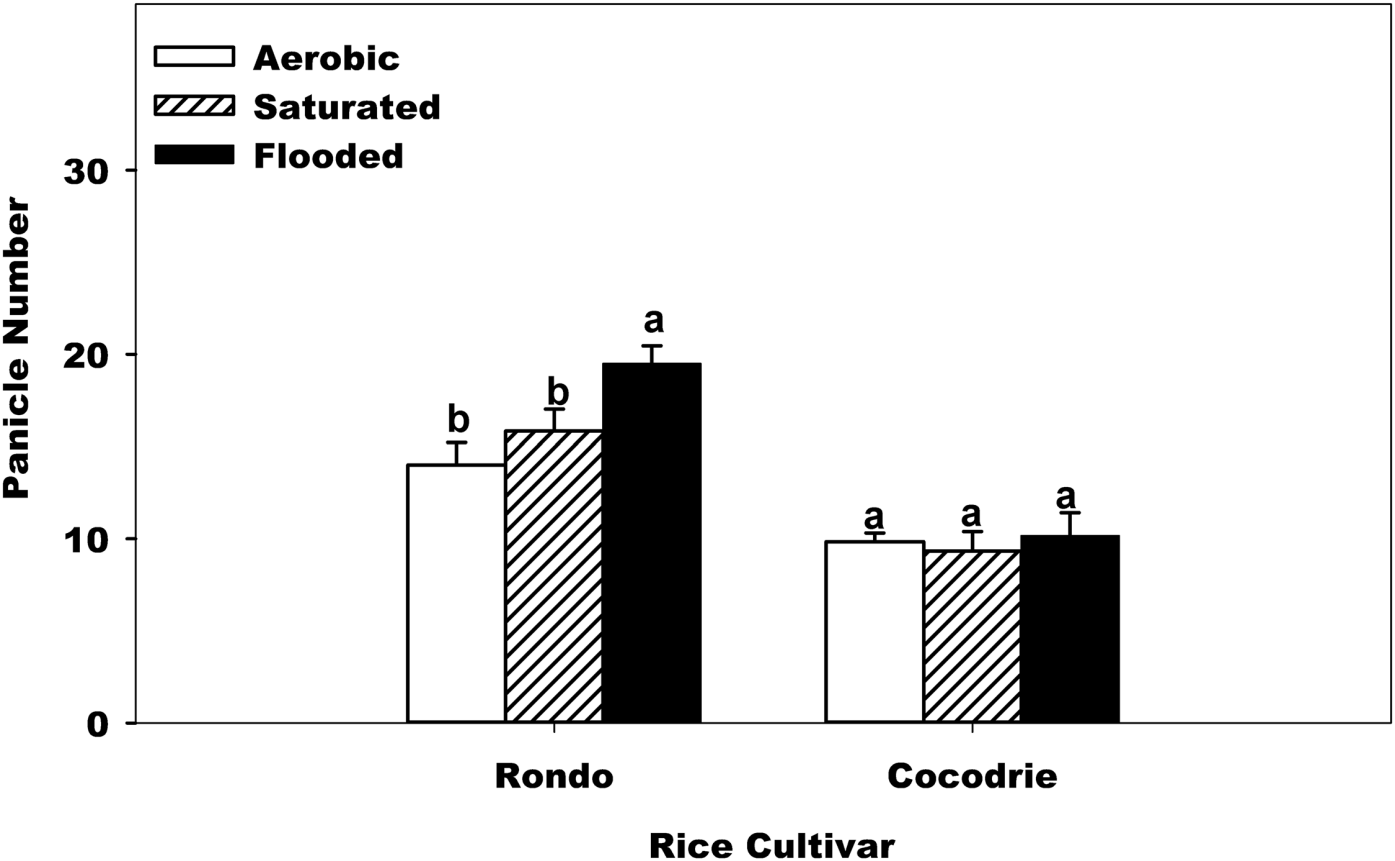
Effect of water regime and cultivar across soil texture on panicle number. Error bars denote the standard error of the mean. Bars with the same letters above are not significantly different based on Fisher’s protected LSD (*P* = 0.05) within the cultivar.

Similar to grain yield, the interaction between water regime and cultivar was found highly significant for panicle number (Table 2). Rondo had decreasing number of panicle from aerobic, to saturated and flooded water regimes. Cocodrie, however, had nearly the same number of panicles in all three water regimes evaluated (Fig 2). These results suggest that Cocodrie had stable productive tiller count and can be grown in both flooded and non-flooded condition, unlike Rondo that needs a flooded condition to produce more productive tillers. Gene mapping study showed a QTL called *WFR* (Wealthy Farmer’s Panicle) [27] encodes OsSLP14 that controls shoot branching in the vegetative stage of rice. Introduction of this QT allele into ‘Nipponbare’ resulted in increased rice production. This QTL could be present in Cocodrie, making it more stable than Rondo in producing productive tillers (having panicle).

Soil texture also significantly affected rice panicle number (Table 2). Panicle number in clay soil was 25% higher than in sandy loam soil (Table 2). Similar to this result, Zhang et al. [28] reported that panicle numbers under clay soil was greater than under sandy soil across nitrogen rate treatments. Bond et al. [29], however, reported that soil texture did not affect rice panicle density. The differences in response to soil texture may be affected by nutrient supply or nutrient uptake of rice which affects rice development and panicle number.

### Number of Spikelet

**Fig 3:**
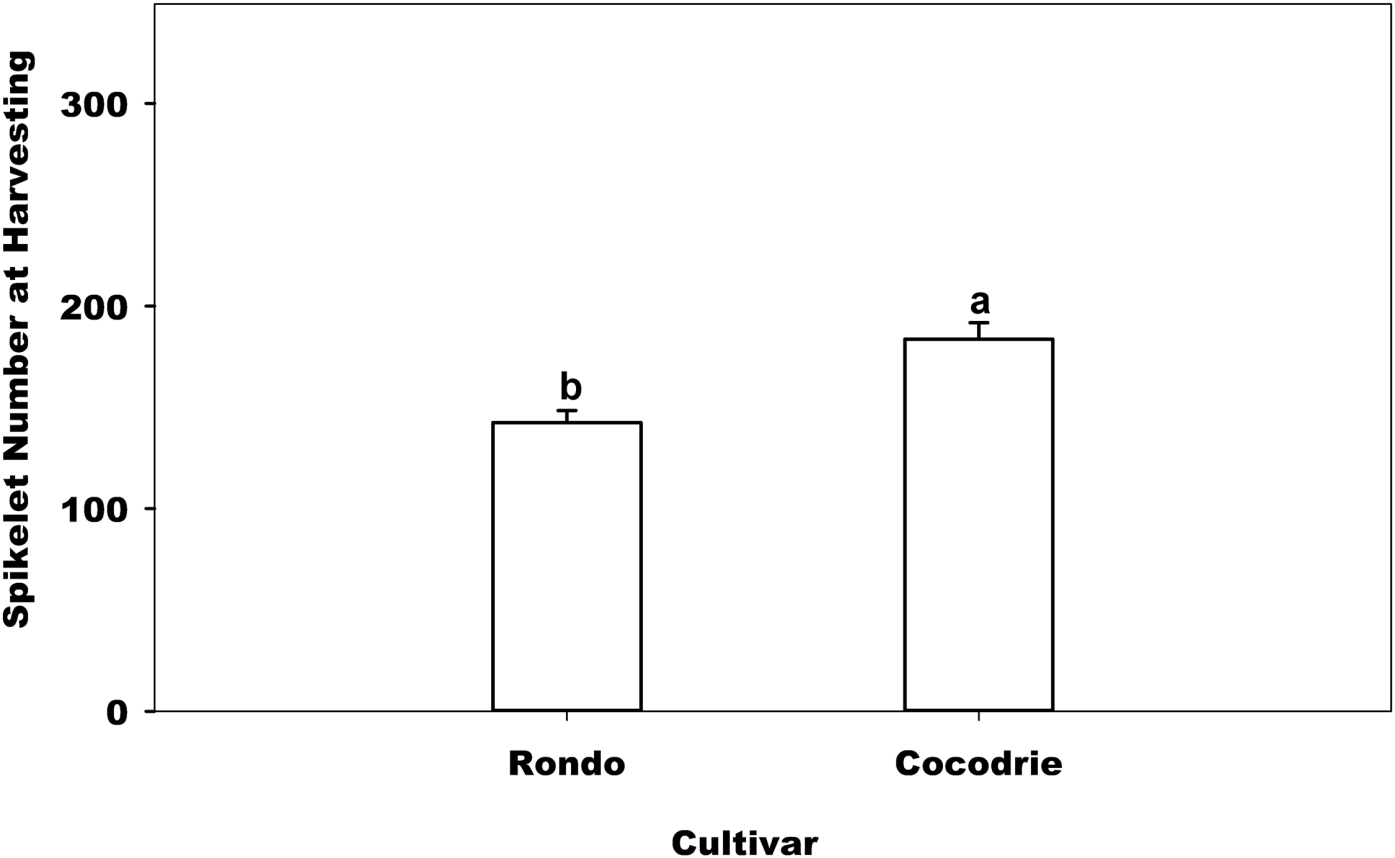
Effect of cultivar on spikelet number at harvesting. Error bars denote the standard error of the mean. Bars with the same letters above are not significantly different based on Fisher’s protected LSD (*P* = 0.05) within the cultivar.

Spikelet number per pot was significantly affected by soil texture (Table 2). Clay soil had 19% greater total spikelet number than sandy loam soil (Table 3). Similar effects of soil on the number of spikelet per panicle have been reported by Rao et al. [30]. Using four different soils, Rao et al. [30] reported that the spikelet per panicle ranged from 43 to 198 varying with soil textures. Those authors partially contributed such variation to the difference in soil boron concentration. Also, the rice cultivar significantly affected the spikelet number (Table 2). The spikelet number of Cocodrie was 29% greater than Rondo, indicating that rice cultivar (genetic) seems to have greater effect on spikelet number than soil condition (Fig 3). QTLs have been reported for spikelet number in rice [31] but being a quantitative trait, environment can still influence its expression. The average grain yield of Rondo was greater than with Cocodrie indicating that greater spikelet per panicle, being one of the yield components, does not necessarily mean greater yield. Such pattern was consistent with the result of Yan et al. [15].

**Fig 4:**
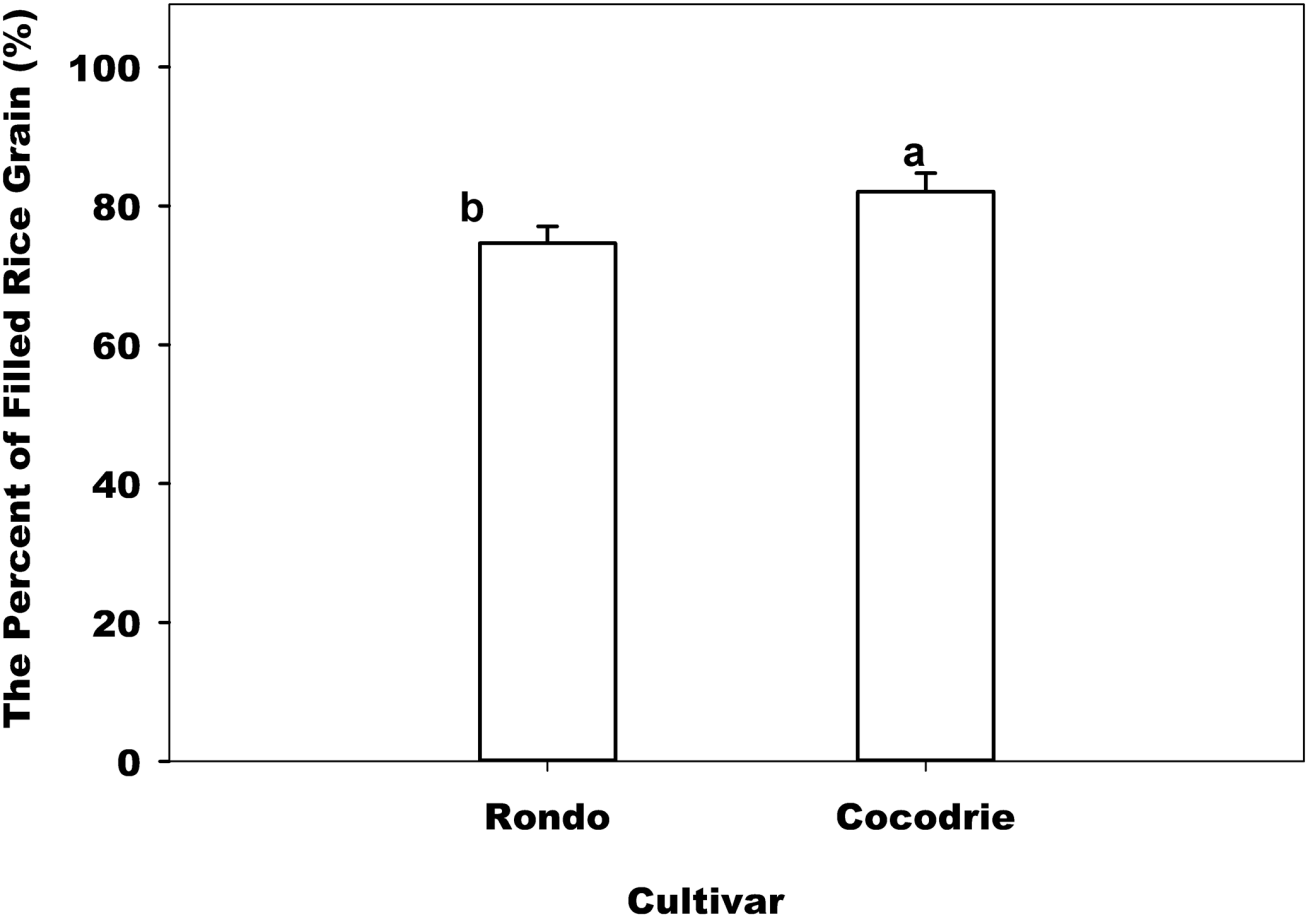
Effect of cultivar on the percentage of filled spikelet number at harvesting. Error bars denote the standard error of the mean. Bars with the same letters above are not significantly different based on Fisher’s protected LSD (*P* = 0.05).

Water regimes did not significantly affect the number of spikelet (Table 2). The same result was noted in regional studies between flooded and non-flooded (ground cover) treatments across 36 sites [32]. Numerically, however, continuous flood had 8% greater number of spikelet (Fig 4) than aerobic water regime which was consistent with the result of Yan [15]. In addition, the number of spikelet per panicle had a big variance across treatments.

### The Percentage of Filled Spikelet

The percentage of filled spikelet was only significantly affected by cultivar (Table 2). Compared to Rondo, Cocodrie had 10% greater percentage of the filled spikelet (Fig 4). Ying et al. [33] reported that grain filling was affected by cultivar too. The difference in grain filling between varieties was as high as 25%. Their results also indicated that cultivar with small panicle size generally filled well, whereas the tropical japonica with large panicle size filled poorly. As noted earlier, Rondo had bigger seeds that were not filled well relative to the smaller seeds of Cocodrie, resulting in much lower percentage of filled grains. Soil did not affect the percentage of filled spikelet.

The effects of water regimens on grain filling varied with varieties. For Rondo, the greatest grain filling was with saturated water treatment. The greatest grain filling for Cocodrie was under flooded treatment. These results were the opposite in grain yield presented earlier indicating the role of other yield components in determining grain yield. Overall, the filled spikelet ranged from 64% to 89% which is consistent with the observations of Yan et al. [15]. Those authors reported that the percentage of grain filling was from 63% to 83% with greater under non-flooded treatment compared to flooded treatment. A regional survey has also reported the similar result that greater percentage of grain filling was with a ground cover treatment (a non-flooded treatment) than a flooded treatment [32].

### Water Productivity

**Fig 5:**
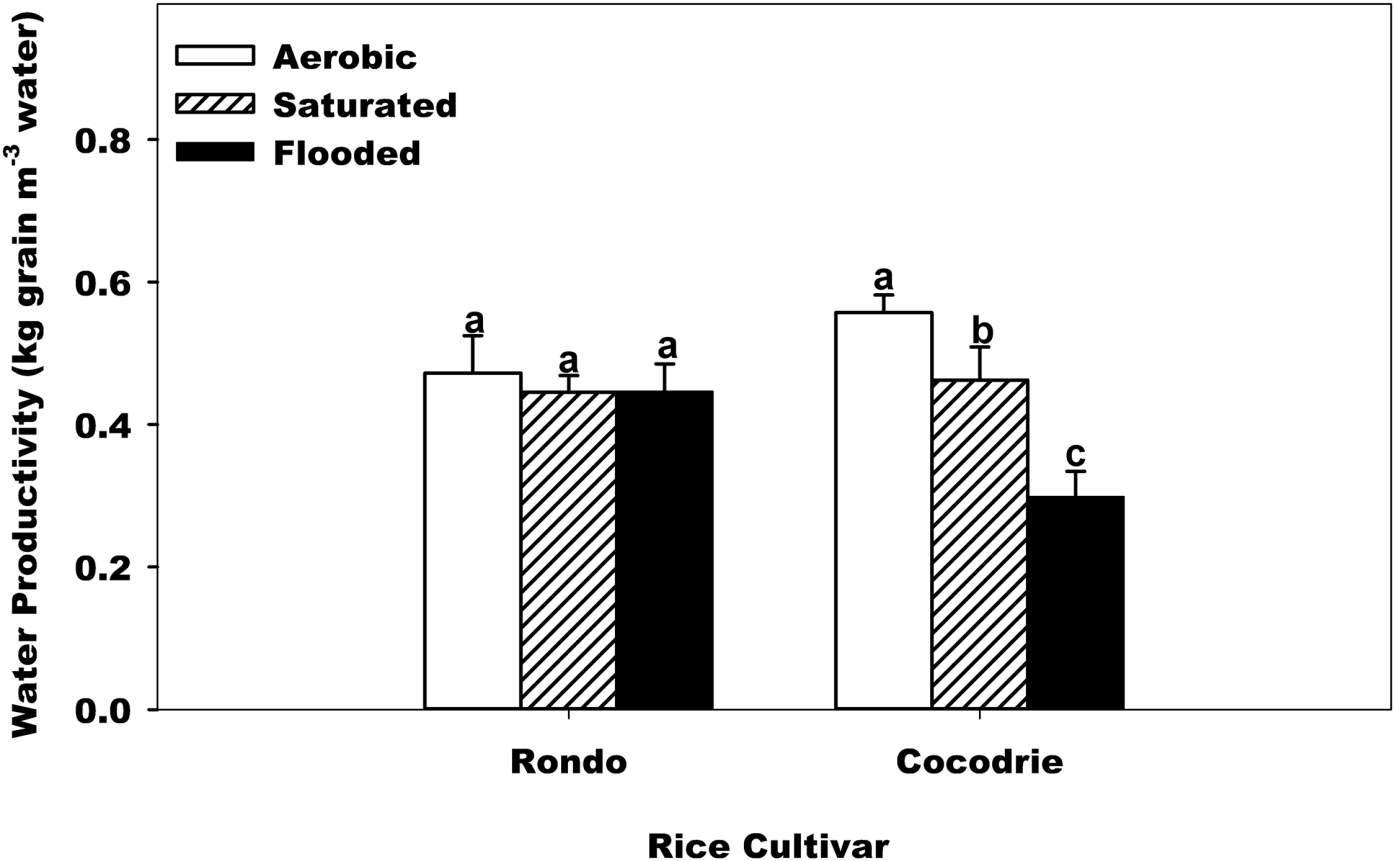
Effect of water regime and cultivar across soil texture on the water productivity (kg grain m^-3^ water). Error bars denote the standard error of the mean. Bars with the same letters above are not significantly different based on Fisher’s protected LSD (*P* = 0.05) within the cultivar.

Water regime and cultivar had a significant interaction effect on water productivity (Table 2). For Cocodrie, the highest water productivity was with aerobic treatment and the lowest with flooded (Fig 1). For Rondo, the water productivities were similar for the water regimes. The overall water productivity ranged from 0.30 to 0.56 kg grain m^-3^ (Fig 5). This result indicated that in limited water production system, even when the cultivar was not selected for aerobic production system, aerobic system is still a practical choice, getting the value for each water drop to produce food. Wang et al. [34] reported the same results that aerobic rice yielded significantly less than lowland rice under fully flooded conditions but its water use was about 60% less and the total water productivity was 1.6 to 1.9 times higher. Water productivity in this experiment is comparable with the report by Tuong [35] regarding the best performing aerobic rice experiments with a water productivity of around 0.5 kg grain m^-3^ water. Also, soil texture had a significant effect on water productivity (Table 2). Compared to the sandy loam soil, clay soil had 25% greater water productivity (Table 3).

Earlier studies on water productivity in aerobic production system were evaluating rainfed or lowland irrigated rice varieties and these were all not selected for aerobic production system, thus inferior yield response was always reported. Considering the low yield and not the high water productivity, aerobic system was not appealing to farmers. Adoption of the technology has been challenging. However, most recent studies as presented earlier using genotypes bred for this system showed comparable or higher yield than flooded system. With foreseeing drought and limited water supply, the benefits of high water productivity and better grain yield of new varieties for aerobic production system will be realized.

## Conclusion

Water regime, soil texture, cultivar and the interaction between water regime and cultivar significantly affected rice production and grain yield. Rondo was best in flooded field but Cocodrie can equally produce grain in the aerobic, saturated and flooded soils. The interaction between water regime and cultivar support the need for water regime specific rice cultivar. Soil texture can influence grain yield. Clay soil that is favorable in retaining water and nutrients than sandy soil is desirable in obtaining higher grain yield. Yield components were generally affected by water regime, cultivar and soil texture except for rice 500-grain weight that was only affected by cultivar. Clay soil produced more tillers and grains, heavier seeds and better grain filling relative to sandy soil. Likewise, flooding had the best among yield components. The two varieties differed in yield components and the desirable combination of these traits determines grain yield. Water productivity varies also with cultivar, water regime and soil texture. Rondo, clay soil and aerobic production system were all efficient in using the applied water compared to Cocodrie, sandy soil and flooded system, respectively. These results indicated that cultivar selection and soil condition are important factors in deciding what water management option to practice, and these have ramification in the field decision process. However, field trials have to be conducted to determine the real field response and the degree of the factors' effect on growth and development. Farmers have favorites too in varieties, and the way they farm in specific location. They usually keep the production practice until they replace old varieties or technology. This could be related to the fact that they have perfected the good combination of cultivar and irrigation in that parcel of land, determined by the type of soil.

## Supporting Information

S1 File Support data of this study. (XLS)
